# Histidine alleviates copper-induced intestinal inflammation through oxidative stress-mediated mitochondrial damage and the Hif1α-Tnfα/Il1β pathway

**DOI:** 10.3389/fimmu.2026.1960076

**Published:** 2026-10-05

**Authors:** Chongchao Zhong, Fang Chen, Shaoxia Lu, Shicheng Han, Hongbai Liu, Chang’an Wang

**Affiliations:** Key Laboratory of Aquatic Animal Diseases and Immune Technology of Heilongjiang Province, Heilongjiang River Fisheries Research Institute, Chinese Academy of Fishery Sciences, Harbin, Heilongjiang, China

**Keywords:** Cu, Hif1α-Tnfα/Il1β pathway, histidine, intestinal inflammation, oxidative stress

## Abstract

**Introduction:**

Copper (Cu) is an essential trace element for vertebrates, but excessive intake can induce intestinal toxicity. Whether nutritional intervention can alleviate Cu overload-induced intestinal damage and the underlying mechanisms remain unclear. In this study, we combined *in vivo* animal experiments with *in vitro* cell models to investigate the protective effect of histidine against Cu-induced intestinal inflammation and its molecular mechanisms.

**Methods:**

Rainbow trout (weight: 7.24 ± 0.03 g) were fed diets containing 3.11 mg Cu/kg and 6.89 g His/kg (Control), 12.69 mg Cu/kg and 6.71 g His/kg (Cu), 3.24 mg Cu/kg and 10.94 g His/kg (His), 12.37 mg Cu/kg and 10.87 g His/kg (Cu+His) for 8 weeks. Intestinal epithelial cells (IECs) were isolated from rainbow trout and incubated for 24-h in Cu (10 μM) and His (2.5 mM) solution. Histopathology, ultrastructural structure, oxidative stress, mitochondrial damage, and inflammation were determined in the intestine and IECs; fluorescence staining (DCFH-DA and JC-1), immunoblotting, immunofluorescence, siRNA, and ChIP were performed on IECs.

**Results:**

Our research results indicate that Cu can induce intestinal oxidative stress, which in turn leads to mitochondrial dysfunction and activation of the Hif1α-Tnfα/Il1β signaling axis, ultimately exacerbating intestinal inflammatory responses. In contrast, co-treatment with Cu and histidine markedly attenuated the intestinal inflammatory phenotype. Mechanistically, histidine exerts anti-inflammatory effects by inhibiting oxidative stress, improving mitochondrial function and suppressing Hif1α, thereby preventing the occurrence of intestinal inflammation.

**Discussion:**

This study is the first to elucidate the mechanism by which histidine alleviates Cu-induced intestinal inflammation and delineated the critical regulatory role of mitochondrial damage and the Hif1α-Tnfα/Il1β pathway in this process within a fish model. It provides a new theoretical basis for mitigating heavy metal toxicity through nutritional intervention.

## 1 Introduction

Copper (Cu) is an essential trace element for vertebrate life, playing an irreplaceable core physiological role in maintaining redox homeostasis, regulating mitochondrial energy metabolism, and mediating immune system functions (1, 2). The intestine, as the central organ for nutrient digestion and absorption, relies heavily on adequate micronutrient supply and a sophisticated immune regulatory network to maintain its microenvironmental homeostasis (3). In recent years, the critical regulatory role of nutritional factors in intestinal health maintenance and disease pathogenesis has become a research hotspot in the field of nutritional immunology (4). Notably, the potential harm of excessive dietary Cu intake to intestinal health is increasingly recognized, with a growing body of evidence indicating a close association between Cu excess and the onset and progression of intestinal inflammatory responses (5, 6). Existing research demonstrates that a Cu excess diet can lead to decreased antioxidant defense capacity and impaired immune function (7). Concurrently, Cu excess compromises the integrity of the intestinal epithelial barrier and increases intestinal permeability (5). Animal studies have further confirmed that Cu excess significantly exacerbates the pathophysiological progression of experimental colitis (6). However, the precise molecular mechanisms underlying Cu excess diet-induced aggravation of intestinal inflammation remain incompletely elucidated. Consequently, strategies for preventing intestinal inflammation and nutritional intervention schemes based on the regulation of Cu homeostasis remain critical scientific questions urgently requiring in-depth exploration in the current field of nutritional immunology.

Histidine, similar to Cu, is an essential nutrient for all vertebrates, including fish (8, 9). Histidine plays important roles in protein structure, enzymatic catalysis, and cellular regulation, indicating its significance in various cellular functions (10). Moreover, changes in histidine levels can also influence various critical physiological processes, including intestinal absorption, immune regulation, and antioxidant defense systems (11). As early as 2009, Andou et al. demonstrated that dietary supplementation with histidine could improve intestinal health (8). Other studies have shown that in the intestinal system of largemouth bass, histidine can suppress intestinal inflammation by enhancing antioxidant capacity (11). Recent research has also revealed that histidine affects intestinal Cu absorption, further supporting the relationship between Cu and histidine in the intestine (12, 13). Given the crucial role of the interaction and homeostasis between histidine and Cu in maintaining intestinal health, this study aims to investigate whether histidine can alleviate intestinal inflammatory responses induced by Cu excess.

Oxidative stress is a regulatory mechanism that plays a key role in modulating inflammation in the body (14). Alterations in intracellular Cu levels can trigger inflammatory responses, a process potentially involving Cu-dependent activation of oxidative stress mechanisms, including changes in Cu-mediated antioxidant enzyme activity and the Fenton reaction (2, 15). Cu excess can exacerbate intestinal oxidative stress, thereby promoting intestinal inflammation through oxidative damage (16). In contrast, histidine can suppress oxidative stress, alleviating intestinal inflammation and promoting intestine health (17). However, the specific interactions between oxidative stress, Cu, and the effects of histidine on intestinal inflammation remain unclear.

Hypoxia-inducible factor-1α (HIF1α), as a central transcription factor for cellular adaptation to hypoxia, plays a crucial role in maintaining intestinal barrier integrity, regulating immune responses, and controlling oxidative stress (18). The functions of HIF1α extend beyond its response to hypoxia and are deeply involved in the pathogenesis of inflammatory bowel disease and metabolic disorders (18). Its subcellular localization, particularly nuclear translocation, is a critical step determining its transcriptional activity (19). This process is tightly regulated by the prolyl hydroxylase domain-containing protein 2 (PHD2)-mediated proteasomal degradation pathway (20). Our recent study showed that Hif1α participates in physiological processes within fish cells and is essential for maintaining normal cellular function (19). Concurrently, the research has demonstrated that Hif1α influences inflammation-related target genes in both fish and mammals and modulates intestinal inflammation by altering the activity of inflammatory factors (21, 22). Existing studies suggest that Cu and histidine may affect the ability of HIF1α to regulate its target genes by influencing oxidative stress (23, 24). In an earlier study from 2008, Shah et al. found that increased HIF1α expression was associated with more severe intestinal inflammatory damage (25). Recent research has also shown that Cu can promote the nuclear translocation of Hif1α, consequently influencing the expression of downstream target genes (26). Nevertheless, the regulatory mechanisms underlying Hif1α involvement in intestinal inflammation under conditions of dietary Cu and histidine supplementation remain poorly understood.

Teleost fish represent the most speciose group of vertebrates. Their intestinal immune regulation exhibits species-specific differences compared to mammals, while maintaining high conservation of core signaling pathways (27, 28). Comparative immunogenomic studies confirm that key functional genes and signaling pathways regulating intestinal inflammation in mammals possess highly conserved orthologs and similar regulatory mechanisms in teleosts (29). Rainbow trout (*Oncorhynchus mykiss*), a globally cultivated cold-water salmonid, serves as a robust experimental model for investigating intestinal inflammation in fish, owing to its well-characterized genome, well-documented intestinal pathology, and functionally validated core inflammatory genes (30, 31). Studies demonstrate that factors like dietary anti-nutritional factors or imbalanced feed formulations can induce significant intestinal mucosal damage, inflammatory responses, and impair barrier function and growth performance in rainbow trout (32–34). However, the mechanisms by which different nutritional or environmental stressors trigger intestinal inflammation, as well as the interactions among key targets, remain incompletely characterized. Building upon previous research on rainbow trout intestinal immunity, this study investigates whether and how histidine supplementation alleviates dietary Cu excess-induced intestinal inflammation.

## 2 Materials and methods

### 2.1 Ethics statement

Formal approval (20250815-002) for all rainbow trout farming protocols in this study was granted by the Committee for the Welfare and Ethics of Laboratory Animals of Heilongjiang River Fisheries Research Institute, CAFS, in accordance with its established animal research ethics standards and China’s ethical guidelines for laboratory animals.

### 2.2 Animals feeding, management

The dietary formulations and their nutritional profiles are detailed in **Supplementary Table 1**. The feeding trial employed a 2×2 factorial arrangement, evaluating two Cu supplementation levels (0.008 and 0.04 g/kg diet, as CuSO4·5H2O, Sinopharm Chemical Reagent Co., Ltd, ShangHai, China, 10008218) and two His levels (4 or 8 g/kg diet, as L-Histidine, Aladdin, ShangHai, China, L425627). This resulted in four experimental groups: Control (0.008 g Cu/kg + 4 g His/kg), Cu (0.04 g Cu/kg + 4 g His/kg), His (0.008 g Cu/kg + 8 g His/kg), and Cu+His (0.04 g Cu/kg + 8 g His/kg). Analysis of the diets yielded the following Cu and His concentrations: Control: 3.11 mg Cu/kg, 6.89 g His/kg; Cu: 12.69 mg Cu/kg, 6.71 g His/kg; His: 3.24 mg Cu/kg, 10.94 g His/kg; Cu+His: 12.37 mg Cu/kg, 10.87 g His/kg. As established in references (35, 36), the Control diet met the Cu and His requirements for rainbow trout.

Throughout the 8-week experimental period, all diets were maintained frozen at -20 °C to preserve integrity. Fresh feed was weighed daily prior to rationing. Fish were reared in a recirculating aquaculture system (RAS) under hygienic conditions. Health status and physiological well-being were monitored daily.

### 2.3 Fish and feeding trial

Juvenile rainbow trout were obtained from a commercial hatchery in Yingkou, China. Prior to the experiment, fish were acclimated to laboratory conditions in 300-L indoor tanks (40 fish per tank) for a two-week period following standardized procedures (37). Subsequently, 300 healthy fish (initial body weight: 7.24 ± 0.03 g, mean ± S.D.) were randomly distributed across twelve 300-L tanks at a density of 25 fish per tank. The feeding trial lasted 8 weeks, during which fish were hand-fed twice daily to apparent satiation with the respective experimental diets at a daily ration of 4% body weight. Feed consumption was recorded per tank on a daily basis. Feeding rates were adjusted weekly based on feeding behavior and biweekly biomass determinations after weighing. Tanks were siphoned daily to remove fecal waste, and mortality was monitored and recorded each day.

Water quality parameters were measured twice weekly and remained within acceptable ranges throughout the experimental period. Water samples were collected weekly 30 min prior to the morning feeding for chemical analysis. Inductively coupled plasma optical emission spectrometry (ICP-OES, Optima 8000DV, PerkinElmer, MA, USA) analysis confirmed that background copper concentrations were below the detection limit (< 1 μg/L).

### 2.4 Sample collection and analysis

At the end of the 8-week feeding trial, rainbow trout were sampled following a 24-h fasting period. Fish were euthanized with tricaine methanesulfonate (MS-222, Sinopharm Chemical Reagent Co., Ltd., AE1052101), and intestinal tissues were immediately dissected on ice. Six fish from each tank (18 fish per group) were randomly picked out and the intestines were obtained, and the mRNA and protein expression, enzyme activity, immunofluorescence analysis were performed. Additional intestinal samples collected randomly from each tank were snap-frozen in liquid nitrogen and stored at −80 °C until further analysis.

Cu concentrations in diets and intestinal tissues were determined by ICP-OES according to a previously established method (9). Briefly, freeze-dried samples were digested in a HNO_3_:HClO_4_ mixture (4:1, v/v) at 120 °C for 1 h. The digests were subsequently diluted with ultrapure water, filtered, and subjected to ICP-OES analysis. Dietary histidine content was measured using an amino acid analyzer (Hitachi, Tokyo, Japan, L-8900) as described by Zhang et al. (37).

### 2.5 Culture and treatments of rainbow trout enterocytes

Primary intestinal epithelial cells (IECs) were isolated from rainbow trout and cultured following a previously established protocol (38). The complete growth medium was based on L-15 basal medium supplemented with 10% fetal bovine serum (FBS, Thermo Fisher Scientific), L-glutamine, and a penicillin-streptomycin antibiotic solution (Sigma-Aldrich). All cells were maintained at 18 °C in a temperature-controlled incubator. To examine cellular responses to copper and histidine co-exposure, cultured IECs were co-incubated with CuSO_4_·5H_2_O and L-histidine at designated concentrations. To delineate the involvement of oxidative stress and the Hif1α signaling pathway in Cu/His-induced intestinal inflammation, two reactive oxygen species (ROS) scavengers were employed: N-acetylcysteine (NAC, 5 mM, MedChem Express, NJ, USA, HY-B0215), a general ROS inhibitor, and MitoTEMPO (10 µM, MedChem Express, HY-112879), a mitochondria-targeted ROS scavenger. For loss-of-function studies, Hif1α knockdown was achieved by transfecting cells with specific small interfering RNA (siRNA) oligonucleotides, where the non-targeted siRNA sequence was set as the negative control group. The siRNA sequences targeting rainbow trout Hif1α are provided in **Supplementary Table 2**. All *in vitro* experiments were performed with three biological replicates, each consisting of three technical replicates.

### 2.6 Histopathology and transmission electronic microscopy

Histological and histochemical staining was performed with hematoxylin–eosin (H&E; Nanjing Jiancheng Institute of Bioengineering, D006-1-1), Alcian Blue-Periodic Acid-Schiff (AB-PAS; Nanjing Jiancheng Institute of Bioengineering, D033-1-2), and DCFH-DA (Beyotime Biotech, S0033M). Ultrastructural analysis of intestinal tissue was carried out by transmission electron microscopy (TEM) as described previously (2, 39). Intracellular ROS levels were assessed in DCFH-DA-stained enterocytes. In brief, cells grown in 12-well plates received the designated treatments. Following 30-min incubation with 5 μM DCFH-DA and three PBS washes, fluorescence was visualized immediately using a laser scanning confocal microscope (Leica Microsystems, Wetzlar, Germany). Fluorescence intensity was measured on a CytoFlex Flow Cytometer (Beckman Coulter), and data were analyzed using FlowJo v.10.

### 2.7 Cell viability and enzymatic activities analysis

Cell viability was determined using the cell counting kit-8 (CCK-8) assay, which is based on the water-soluble tetrazolium salt 8-[2-(2-methoxy-4-nitrophenyl)-3-(4-nitrophenyl)-5-(2,4-disulfophenyl)-2H-tetrazolium] monosodium salt, following a standard protocol (9). The activities of Copper/Zinc superoxide dismutase (Cu/Zn-SOD) and catalase (CAT), total antioxidant capacity (T-AOC), and malondialdehyde (MDA) content were measured using commercially available assay kits from the Nanjing Jiancheng Institute of Bioengineering (Jiangsu, China). The corresponding kit catalog numbers are as follows: Cu/Zn-SOD (A001-2-2), CAT (A007-1-1), T-AOC (A015-1-2), and MDA (A003-1-2). Tnfα (FSA0045-01) and Il1β (FSA0003-01) contents were determined with the ELISA kits from Fensbe Bioengineering (Wuhan, China). All assays were performed strictly in accordance with the manufacturer’s instructions.

### 2.8 Determination of ATP, NAD+/NADH, mtDNA, and mitochondrial transmembrane potentials (ΔΨm, MMPs)

Adenosine triphosphate (ATP) and nicotinamide adenine dinucleotide (NAD^+^/NADH) levels were measured using commercial assay kits from the Nanjing Jiancheng Institute of Bioengineering (Nanjing, China, A095-1-1 and A114-1-1, respectively). Mitochondrial DNA (mtDNA) was extracted using a kit from Shanghai Saint-Bio Biotechnology (Shanghai, China, 12021). mtDNA copy number alterations in intestinal tissues and isolated enterocytes were subsequently verified by RT-PCR and qRT-PCR, respectively, following established laboratory procedures (9). Intracellular mitochondrial membrane potential (MMP) was determined using the JC-1 fluorescent probe (Beyotime Biotech, C2006) as described previously (9). Briefly, cells were incubated with 5 μg/mL JC-1 for 30 min at 18 °C, washed three times with phosphate-buffered saline (PBS), and immediately imaged by laser scanning confocal microscopy (Leica Microsystems, Wetzlar, Germany). Fluorescence intensity was quantified using a CytoFlex flow cytometer (Beckman Coulter), and data were analyzed with FlowJo v.10 software.

### 2.9 RNA isolation and qPCR analysis

Total RNA was isolated from rainbow trout intestinal tissue and enterocytes with TRIzol reagent (Thermo Fisher Scientific, 10296028), as previously described (2). Complementary DNA (cDNA) was synthesized from the extracted RNA using a Reverse Transcription Kit (TaKaRa, Tokyo, Japan, 6110A). To ensure accurate quantification, eight candidate reference genes (*tuba, 18s rrna, rpl7, β-actin, hprt, tbp, gapdh*, and *ubce*) were evaluated for expression stability. The most stable pair, selected by the geNorm algorithm, was used for normalization (primer sequences in **Supplementary Table 2**). Relative mRNA levels, expressed as fold change versus controls, were determined by the 2^-ΔΔCt^ method (40), normalized against the geometric mean of the two optimal reference genes.

### 2.10 Immunoblot

Western blot analysis was performed to assess protein expression levels following protocols from our previous studies (9, 39). The following target proteins were examined: Hif1α, Phd2, Histone-H3, and β-actin. Briefly, intestinal tissues and enterocytes were lysed in RIPA buffer (Biosharp, Hefei, China; BL504A) on ice for 30 min. Equal amounts of protein lysates were separated by SDS-PAGE and electrotransferred onto polyvinylidene difluoride (PVDF) membranes (Thermo Fisher Scientific, 88518). Membranes were then blocked with 8% (w/v) non-fat milk in Tris-buffered saline containing Tween 20 (TBST) for 2 h at room temperature, followed by three 5-min washes with TBST. Subsequently, membranes were incubated overnight at 4 °C with the following primary antibodies: anti-Hif1α (1:5000; Proteintech, 20960-1-AP), anti-Phd2 (1:5000; Proteintech, 19886-1-AP), anti-Histone-H3 (1:6000; Proteintech, 17168-1-AP), and anti-β-actin (1:10000; Abclonal, AC026). After washing, membranes were incubated with HRP-conjugated anti-rabbit IgG secondary antibody (1:10000; Cell Signaling Technology, #7074). Protein bands were visualized using a Vilber FUSION FX6 Spectra imaging system (Vilber Lourmat) and quantified with Image-Pro Plus 6.0 software (Media Cybernetics).

### 2.11 Immunofluorescence analysis

Immunofluorescence staining was performed to examine the expression and subcellular localization of Hif1α in rainbow trout intestinal tissues and enterocytes, following a previously established protocol (2). Briefly, tissue sections and cells were incubated with an anti-Hif1α primary antibody (1:200; Proteintech, 20960-1-AP), followed by incubation with ABflo^®^ 594-conjugated goat anti-rabbit IgG (H+L) secondary antibody (1:500; Abclonal, AS039). Nuclei were counterstained with 4′,6-diamidino-2-phenylindole (DAPI; Beyotime Biotech, C1002). Fluorescent images were captured using a Leica laser scanning confocal microscope. Colocalization between proteins was quantified on the resulting images using the Manders algorithm and ImageJ software (NIH, USA) with the fiji plug-in as previously described (41).

### 2.12 Chromatin immunoprecipitation

ChIP assays were performed on rainbow trout enterocyte DNA as described by Zhong et al. (2). Briefly, following 24 h of Cu and histidine co-treatment, cells were fixed with 1% formaldehyde for 10 min at 37 °C to cross-link protein-DNA complexes. Cross-linking was quenched by adding 1× glycine for 5 min, and cells were then washed with PBS containing 1 mM PMSF. Cell lysis was carried out in SDS lysis buffer supplemented with protease inhibitors, and chromatin was sheared to 200–1000 bp fragments by sonication. After removing cellular debris by centrifugation, the chromatin supernatant was diluted with ChIP dilution buffer. Immunoprecipitation was performed using an anti-Hif1α antibody (Proteintech, 20960-1-AP) and a ChIP Assay Kit (Beyotime Biotech, P2078). Protein-bound magnetic beads were then washed sequentially with low-salt, high-salt, LiCl immune complex, and TE buffers. Cross-links were reversed by incubation in elution buffer containing 200 mM NaCl for 4 h at 65 °C, and the purified DNA was recovered using an AxyPrep PCR Clean-Up Kit (Axygen, AP-PCR-250). Precipitated DNA fragments were quantified by qPCR with primers listed in **Supplementary Table 2**.

### 2.13 Statistical analysis

All data are presented as mean ± standard deviation (S.D.). Statistical analyses were performed using SPSS 22.0 software (IBM, Armonk, NY, USA). One-way analysis of variance (ANOVA) with Duncan’s post-hoc test was performed for levels screening of Cu, histidine and the test drug, whereas the Student’s t-test was used for pairwise comparisons between two groups. Differences were considered statistically significant at *P* < 0.05. Graphical representations were generated using GraphPad Prism 8.0.

## 3 Results

### 3.1 *In vivo* studies

#### 3.1.1 Histidine alleviates growth performance impairment in rainbow trout caused by dietary Cu excess

Given the critical roles of Cu and His in growth metabolism, we investigated their individual and combined effects on these parameters in rainbow trout. Fish were fed experimental diets as follows: control (adequate Cu and His), Cu, His, and Cu+His. Dietary Cu and His levels did not affect initial mean body weight (IBW) or Survival (**Table 1**). Notably, compared with the control group, the Cu group significantly reduced the final average body weight (FBW), weight gain (WG), and specific growth rate (SGR). Conversely, FBW, WG, and SGR of the Cu + His group were significantly higher than those of the Cu group. These findings collectively indicate that dietary Cu excess impairs rainbow trout growth performance, while histidine supplementation alleviates the adverse effects of Cu excess on growth.

**Table 1:** Growth performances of rainbow trout (mean ± S.D, n = 3) fed experimental diets for 8 weeks*.

Parameter	Control	Cu	His	Cu+His
IBW (g/fish)	7.23 ± 0.04	7.23 ± 0.01	7.26 ± 0.03	7.25 ± 0.04
FBW (g/fish)	27.43 ± 1.30	21.90 ± 1.25^*^	27.74 ± 1.56	26.25 ± 1.16^#^
WG (%)	279.39 ± 20.02	202.81 ± 17.59^*^	283.64 ± 22.41	262.14 ± 14.23^#^
SGR (%/d)	2.38 ± 0.10	1.98 ± 0.10^*^	2.40 ± 0.11	2.30 ± 0.07^#^
Survival (%)	100.00 ± 0.00	100.00 ± 0.00	100.00 ± 0.00	100.00 ± 0.00

*Values are means ± S.D. ^*^P < 0.05 compared with the Control-treated group. #Values are means ± S.D. ^#^*P* < 0.05 compared with the Cu-treated group.

IBW (g/fish) = initial mean body weight;

FBW (g/fish) = final mean body weight;

WG (weight gain, %) = (FBW − IBW)/IBW × 100;

SGR (specific growth rate, %/d) = (Ln FBW − Ln IBW)/days × 100;

Survival (%) = final fish number/initial fish number × 100.

#### 3.1.2 Histidine alleviates intestinal inflammation caused by dietary Cu excess in rainbow trout

Histological analysis revealed severe damage to intestinal villi in the Cu group compared to the control group. Villus morphology was significantly improved in the Cu+His group relative to the Cu group (**Figures 1A, B**). The Cu group exhibited significantly reduced villi length and intestinal wall thickness compared to control group (**Figures 1C–E**). Conversely, the Cu+His group showed significantly increased villi length and wall thickness versus the Cu group (**Figures 1C–E**). Goblet cells number were also significantly decreased in the Cu group compared to control, but significantly increased in the Cu+His group relative to the Cu group (**Figure 1F**).

**Figure 1:**
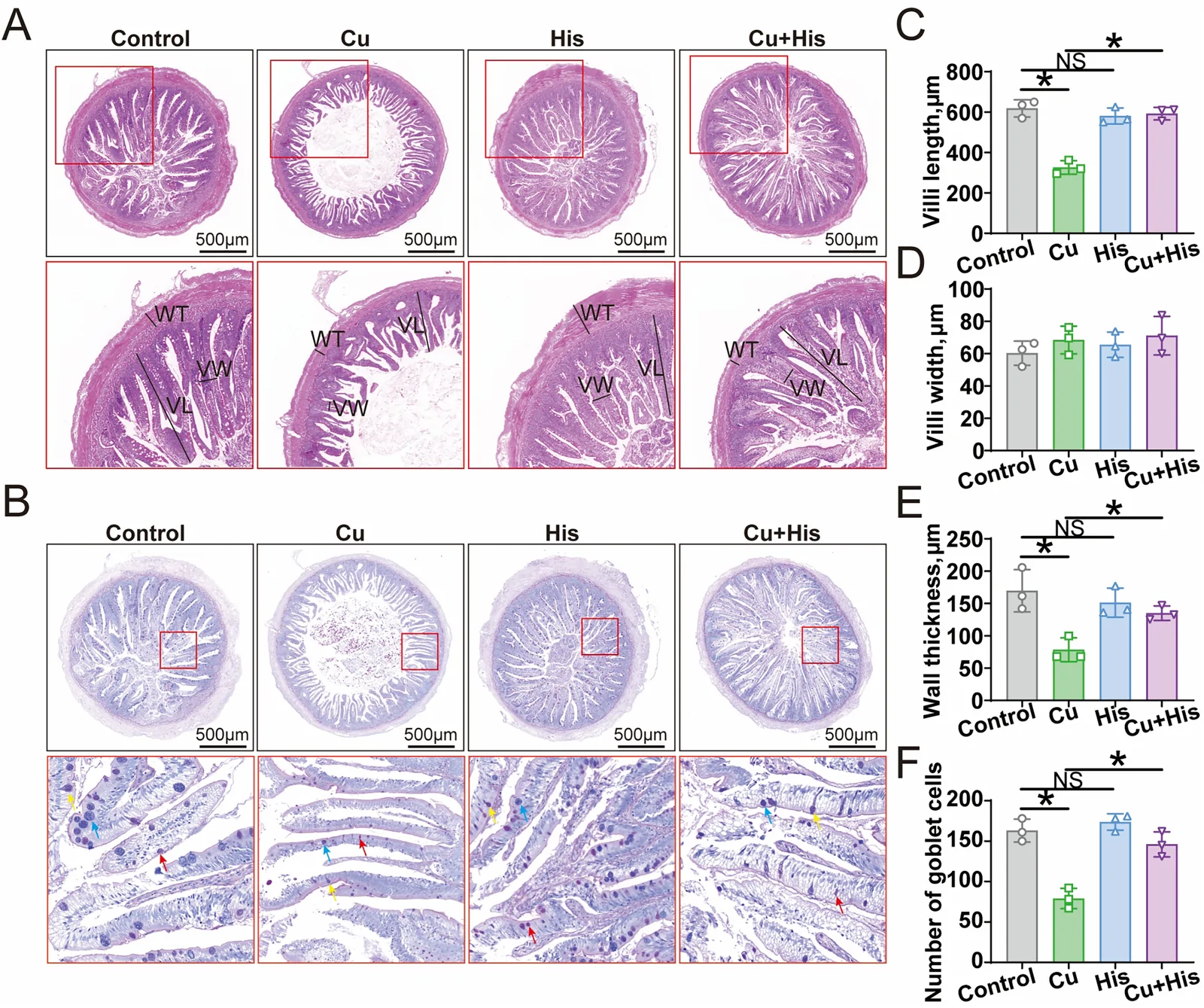
Effects of dietary Cu and His on the intestine health of rainbow trout. **(A)** Representative micrographs of H&E (40 × and 200 × magnification), VL, Villi length; VW, Villi width; WT, Wall thickness. **(B)** Representative micrographs of AB-PAS (40 × and 200 × magnification), red arrow: neutral mucus; blue arrow: acid mucus; yellow arrow: goblet cells. **(C)** Villi length. **(D)** Villi width. **(E)** Wall thickness. **(F)** Number of goblet cells. Values are means ± S.D. (*n* = 3 replicate tanks). *p* value was calculated by Student’s t-tests. Asterisk (*) indicate significant difference among two groups (*p < 0.05).

Furthermore, analysis of Cu content and inflammation-related factors in intestinal tissues. Compared to the control group, the Cu group showed a significant increase in intestinal Cu content (**Figure 2A**). Compared to the control group, the Cu group significantly upregulated the mRNA expression levels of *tnfα*, *il1β*, and *il6* (**Figure 2B**), while the His group significantly downregulated the mRNA expression level of *il8* (**Figure 2B**). Compared to the Cu group, the Cu+His group significantly reduced the mRNA expression levels of *tnfα* and *il1β* (**Figure 2B**). Consistent with mRNA data, intestinal Tnfα and Il1β contents were significantly elevated in the Cu group compared to control, but significantly reduced in the Cu+His group relative to the Cu group (**Figures 2C, D**). Thus, dietary histidine supplementation ameliorated Cu excess-induced intestinal inflammation and morphological damage.

**Figure 2:**
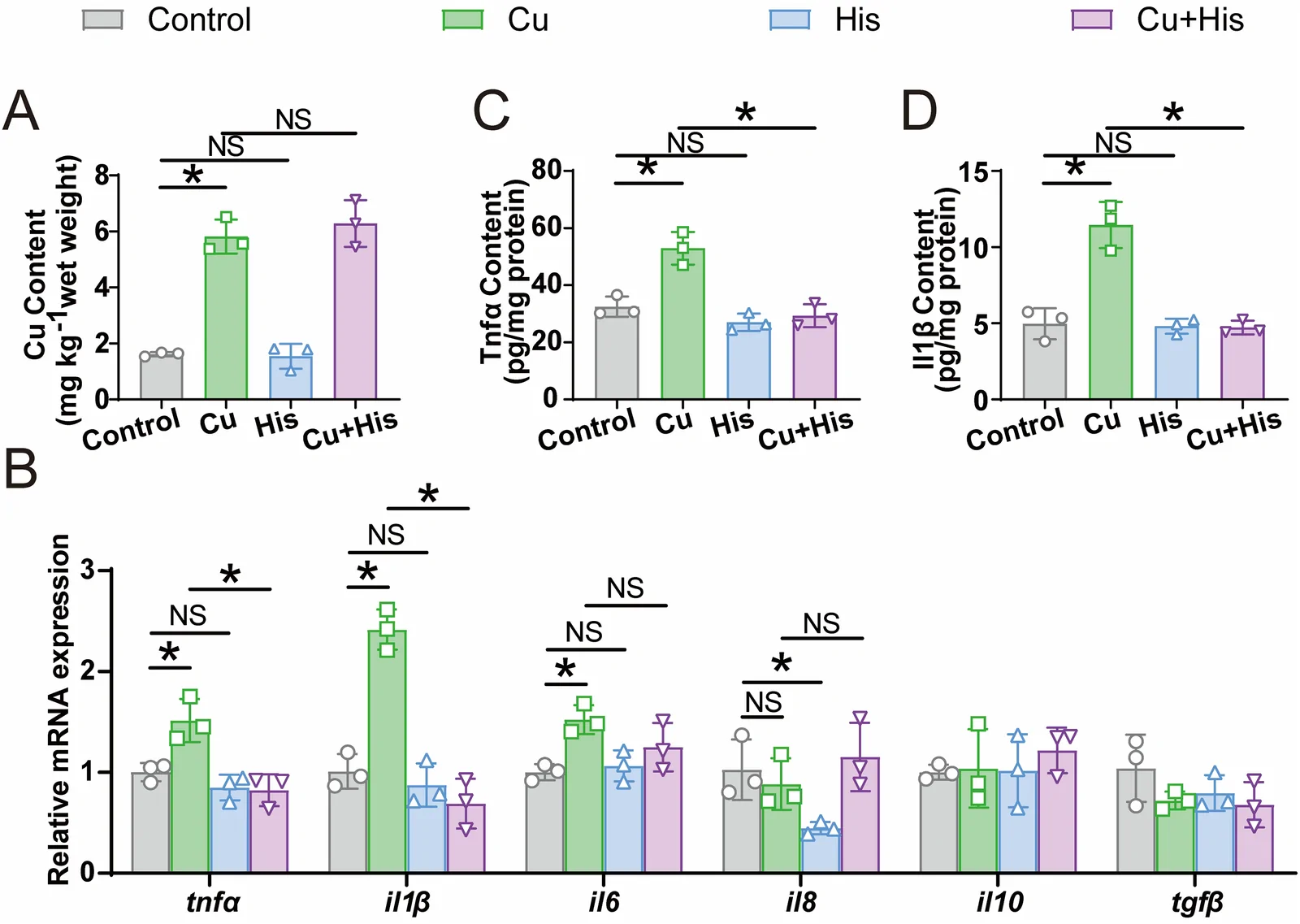
Effects of dietary Cu and His on Cu content and inflammation in the intestine of rainbow trout. **(A)** Cu content. **(B)** The relative mRNA expression of inflammation-related genes. **(C)** Tnfα content. **(D)** Il1β content. Values are means ± S.D. (*n* = 3 replicate tanks and 6 fish were sampled from each tank). *p* value was calculated by Student’s t-tests. Asterisk (*) indicate significant difference among two groups (*p < 0.05).

#### 3.1.3 Histidine alleviates intestinal oxidative stress and mitochondrial damage caused by dietary Cu excess in rainbow trout

Compared to the control, the Cu group exhibited significantly elevated MDA content alongside reduced T-AOC, Cu/Zn-SOD activity, CAT activity, and GSH/GSSG ratio (**Figures 3A–E**). The His group alone significantly increased Cu/Zn-SOD activity versus control (**Figure 3C**). Relative to Cu, the Cu+His group demonstrated significantly decreased MDA levels and elevated T-AOC, Cu/Zn-SOD activity, CAT activity, and GSH/GSSG ratio (**Figures 3A–E**). Gene expression analysis revealed that Cu significantly downregulated *sod1*, *cat*, and *gr* mRNA compared to control (**Figure 3F**). In contrast, histidine supplementation significantly upregulated sod1 expression (**Figure 3F**). Furthermore, Cu+His significantly increased expression of *sod1*, *cat*, and *gr* versus the Cu group (**Figure 3F**).

**Figure 3:**
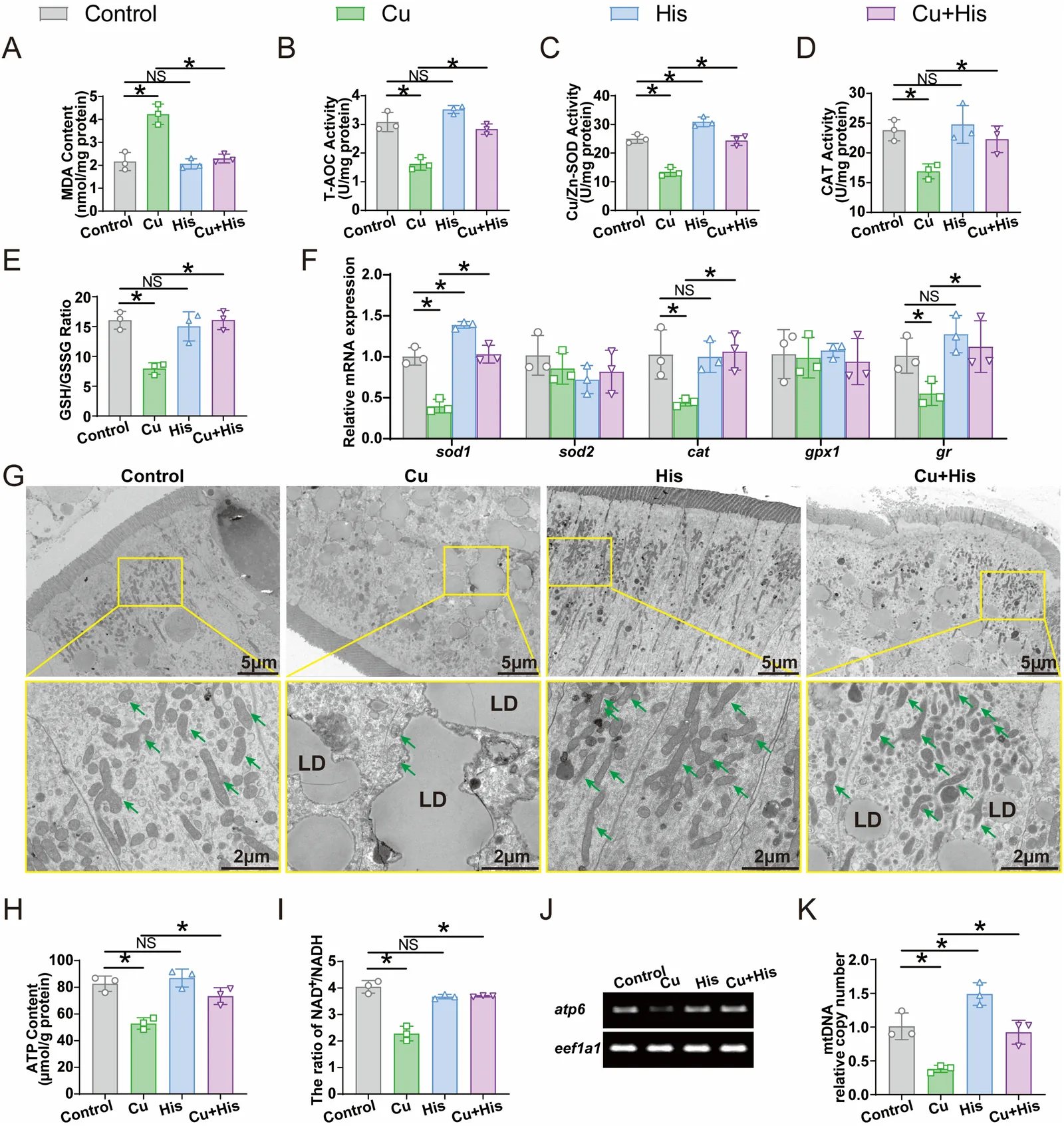
Effects of dietary Cu and His on oxidative stress and mitochondrial function in the intestine of rainbow trout. **(A)** MDA content. **(B)** T-AOC activity. **(C)** Cu/Zn-SOD activity. **(D)** CAT activity. **(E)** GSH/GSSG ratio. **(F)** The relative mRNA expression of antioxidant-related genes. **(G)** Representative TEM image. Green arrows represent mitochondrion; LD: Lipid droplet. **(H)** ATP content. **(I)** NAD+/NADH ratio. **(J)** Characteristics of mitochondrial function-deficient cells, and the expression of mtDNA was analyzed by RT-PCR using *eef1a1* as an internal standard. **(K)** Characteristics of mitochondrial function-deficient cells, and the expression of mtDNA was analyzed by qRT-PCR using *eef1a1* as an internal standard. Values are means ± S.D. (*n* = 3 replicate tanks and 6 fish were sampled from each tank). *p* value was calculated by Student’s t-tests. Asterisk (*) indicate significant difference among two groups (*p < 0.05).

TEM revealed prominent mitochondrial swelling, decreased mitochondrial number, and increased lipid droplets in the Cu group (**Figure 3G**). These pathological alterations were markedly attenuated in the Cu+His group (**Figure 3G**). The Cu group showed characteristics of mitochondrial dysfunction, including decreased ATP content and NAD^+^/NADH ratio (9). However, Cu+His increased both ATP content and NAD^+^/NADH ratio compared to Cu (**Figures 3H, I**). Mitochondrial copy number analysis confirmed a significant decrease in Cu versus control and an increase in His relative to control (**Figures 3J, K**). Moreover, Cu+His displayed increased mitochondrial copy number versus Cu (**Figures 3J, K**). Collectively, these findings indicate that dietary histidine supplementation effectively mitigates Cu excess-induced intestinal oxidative stress and mitochondrial damage.

#### 3.1.4 Histidine alleviates the increase of Hif1α expression into the nucleus caused by dietary Cu excess in rainbow trout

To elucidate the mechanisms underlying intestinal inflammation regulation, we examined the expression and subcellular localization of key oxidative stress-related regulators, Phd2 and Hif1α, in fish intestine. Compared to control, the Cu group exhibited significantly increased *mtf-1* and *hif1α* mRNA expression, alongside decreased *foxo3* and *phd2* expression (**Figures 4A, B**). The His group significantly upregulated *nrf2* but downregulated *hif1α* mRNA compared to control (**Figures 4A, B**). Relative to Cu, the Cu+His group showed significantly reduced *hif1α* and elevated *phd2* mRNA expression (**Figures 4A, B**). At the protein level, Cu significantly upregulated both T-Hif1α and N-Hif1α compared to control, while Phd2 protein expression showed the opposite trend (**Figures 4C, D**). Conversely, Cu+His significantly downregulated T-Hif1α and N-Hif1α while upregulating Phd2 protein compared to Cu (**Figures 4C, D**). Immunofluorescence analysis of intestinal tissue directly confirmed Cu-induced nuclear translocation of Hif1α (**Figures 4E, F**). Critically, this nuclear accumulation was attenuated in the Cu+His group (**Figures 4E, F**). Collectively, these results demonstrate that dietary histidine supplementation mitigates the Cu excess diet-induced increase in nuclear Hif1α expression.

**Figure 4:**
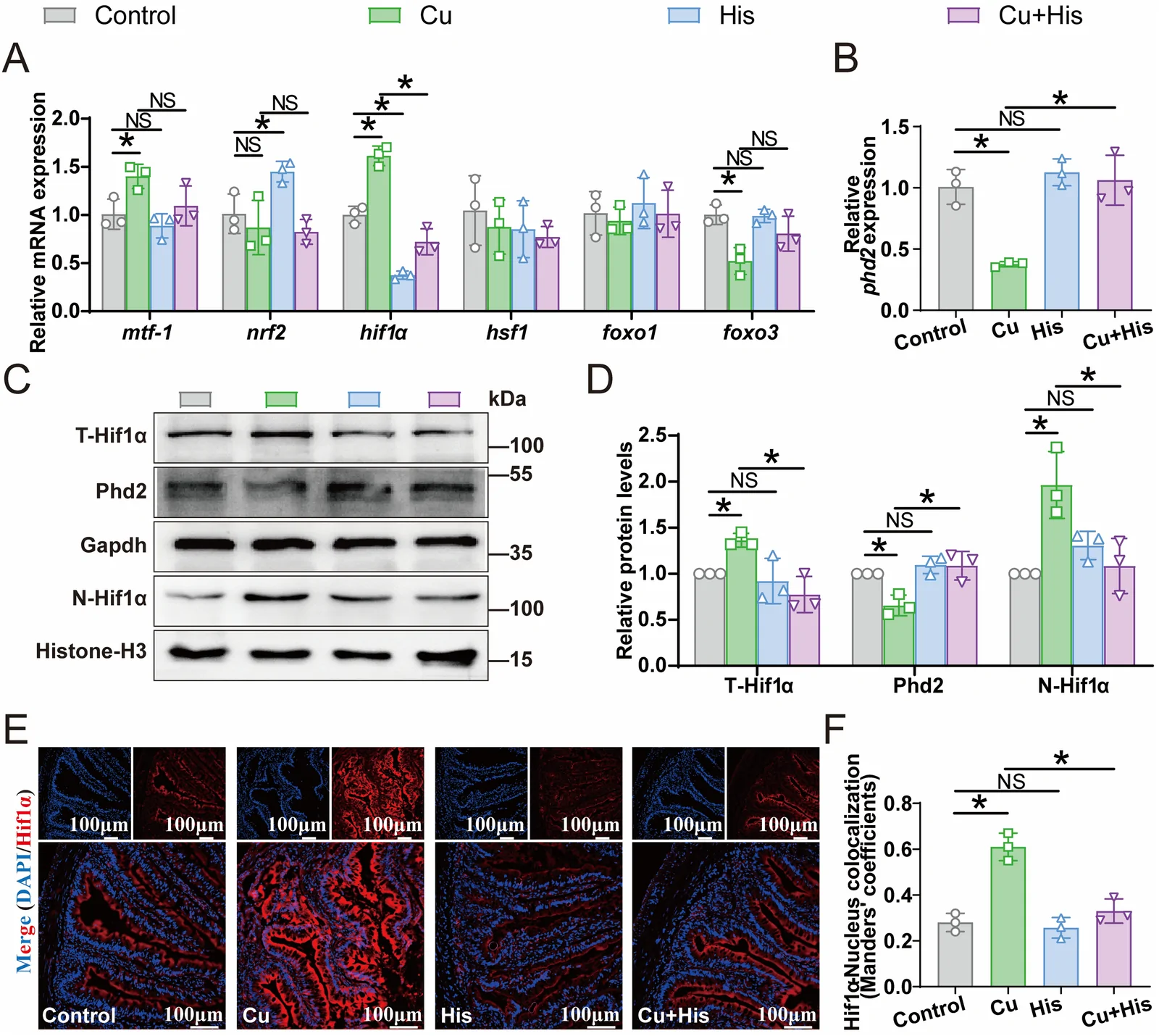
Effects of dietary Cu and His on the Phd2 and Hif1α in the intestine of rainbow trout. **(A)** Relative mRNA expression levels of transcription factor genes associated with oxidative stress. **(B)** *phd2* mRNA expression. **(C, D)** Western blot analysis of T-Hif1α (total-Hif1α), Phd2, and N-Hif1α (nucleus-Hif1α) protein expression. **(E)** Immunofluorescence staining showed the typical confocal image and the relative red fluorescence intensity of Hif1α protein. **(F)** Hif1α-to-Nucleus colocalization was quantified as Manders’ coefficients of images obtained in panel **(E)** Values are means ± S.D. (*n* = 3 replicate tanks and 6 fish were sampled from each tank). *p* value was calculated by Student’s t-tests. Asterisk (*) indicate significant difference among two groups (*p < 0.05).

### 3.2 *In vitro* studies

To further elucidate the mechanisms by which Cu and His influence intestinal inflammation, we conducted a series of *in vitro* experiments. The environment of the *in vitro* cell culture system is significantly different from that of the *in vivo* dietary feeding. Therefore, the levels of Cu and histidine in the dietary cannot be directly used. Thus, we established gradient concentrations of Cu and histidine to assess viability in primary rainbow trout intestinal epithelial cells. Based on CCK-8 assays, this *in vitro* study utilized four groups: Control (no added Cu/histidine), Cu (10 µM Cu), His (2.5 mM histidine), and Cu+His (10 µM Cu+2.5 mM histidine) (**Supplementary Figures 1A–C**).

#### 3.2.1 Histidine alleviates Cu-induced oxidative stress, mitochondrial damage, inflammation, and increased nuclear expression of Hif1α in rainbow trout enterocytes

Compared to the control, the Cu group exhibited significantly elevated MDA content alongside reduced Cu/Zn-SOD, T-AOC, and CAT activities (**Figures 5A–D**). The His group showed increased Cu/Zn-SOD activity relative to control (**Figure 5B**). Conversely, Cu+His demonstrated significantly decreased MDA levels and increased Cu/Zn-SOD, T-AOC, and CAT activities compared to Cu (**Figures 5A–D**). ROS levels were significantly elevated in Cu versus control, while Cu+His significantly reduced ROS levels relative to Cu (**Figures 5E–G**; **Supplementary Figure 4A**).

**Figure 5:**
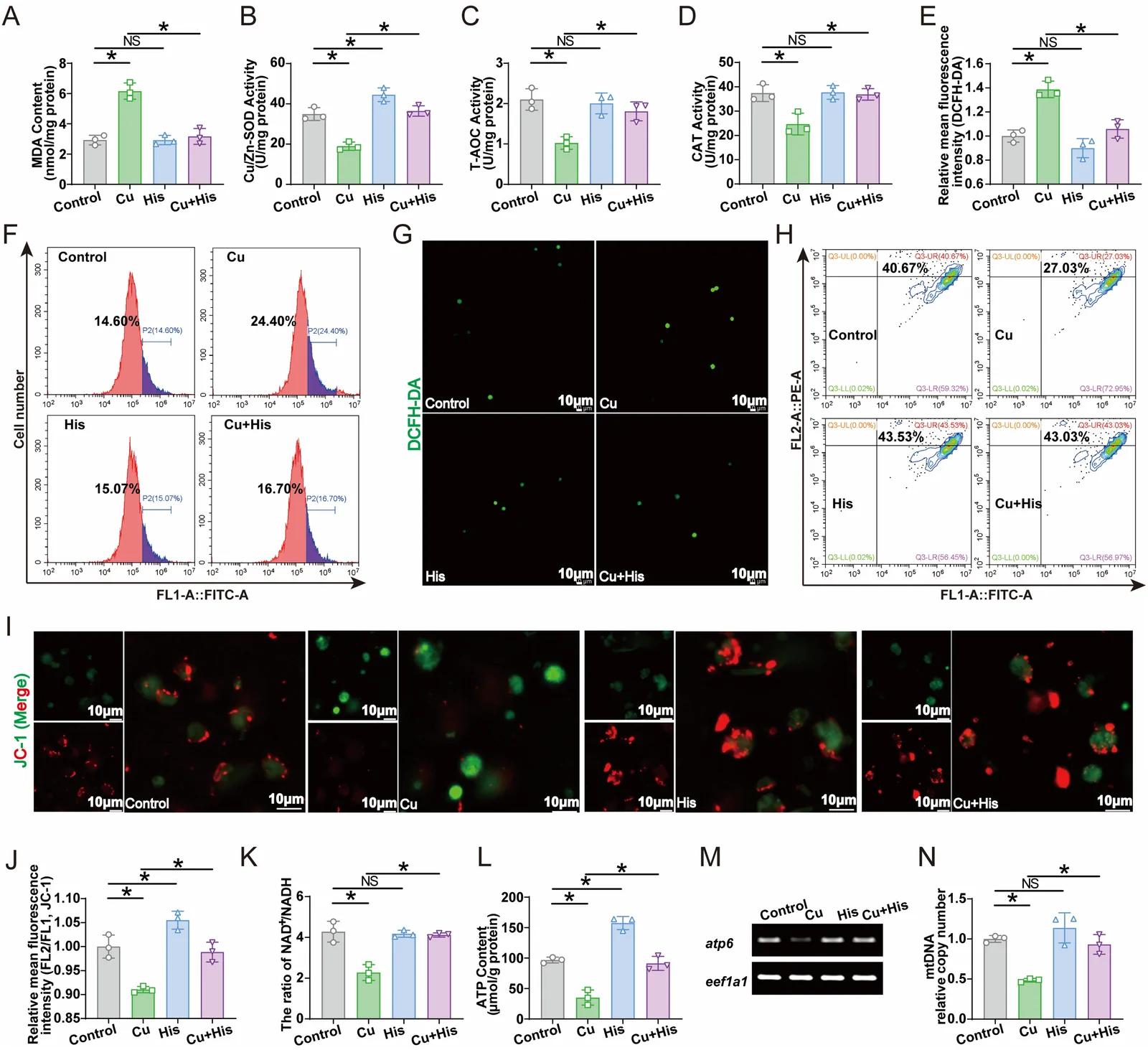
Cu and His regulated oxidative stress and mitochondrial function in rainbow trout enterocytes. Isolated rainbow trout enterocytes were subjected to Cu and His for 24 h. **(A)** MDA content. **(B)** Cu/Zn-SOD activity. **(C)** T-AOC activity. **(D)** CAT activity. **(E)** The intracellular ROS was quantified by calculating FL1 (green) mean fluorescence intensity (DCFH-DA fluorescent staining). **(F)** The fluorescence intensity of ROS was measured by flow cytometry. **(G)** Representative confocal microscopy image of hepatocytes stained with DCFH-DA, Scale bars, 10 μm. **(H)** The presence of JC-1 intracellular mitochondrial transmembrane potential (MMP) was determined by flow cytometry analysis of red/green (FL2/FL1) fluorescence ratio. **(I)** Representative confocal microscopic image of hepatocytes stained with JC-1. Red fluorescence indicates normal ΔΨm with JC-1 aggregates in mitochondria and green fluorescence reflects cytosolic JC-1 monomer indicative of ΔΨm loss. Scale bar, 10 μm. **(J)** The MMP was quantified by flow cytometric analysis of red/green (FL2/FL1) fluorescence ratio (JC-1 fluorescent staining, 5 μM). **(K)** NAD+/NADH ratio. **(L)** ATP content. **(M)** Characteristics of mitochondrial function-deficient cells, and the expression of mtDNA was analyzed by RT-PCR using eef1a1 as an internal standard. **(N)** Characteristics of mitochondrial function-deficient cells, and the expression of mtDNA was analyzed by qRT-PCR using eef1a1 as an internal standard. Values are means ± S.D. (*n* = 3 independently biological experiments). *p* value was calculated by Student’s t-tests. Asterisk (*) indicate significant difference among two groups (*p < 0.05).

Mitochondrial function assessment revealed that Cu significantly decreased JC-1 aggregate formation (indicating membrane potential loss), NAD^+^/NADH ratio, ATP content, and mtDNA copy number compared to control (**Figures 5H–N**; **Supplementary Figure 4B**). The His group showed increased ATP content versus control (**Figure 5L**). Cu+His significantly increased JC-1 aggregates, NAD^+^/NADH ratio, ATP content, and mtDNA copy number relative to Cu (**Figures 5H–N**; **Supplementary Figure 4B**). Regarding inflammation, Cu significantly increased Tnfα and Il1β contents compared to control, whereas Cu+His significantly reduced these cytokine levels relative to Cu (**Figures 6A, B**).

**Figure 6:**
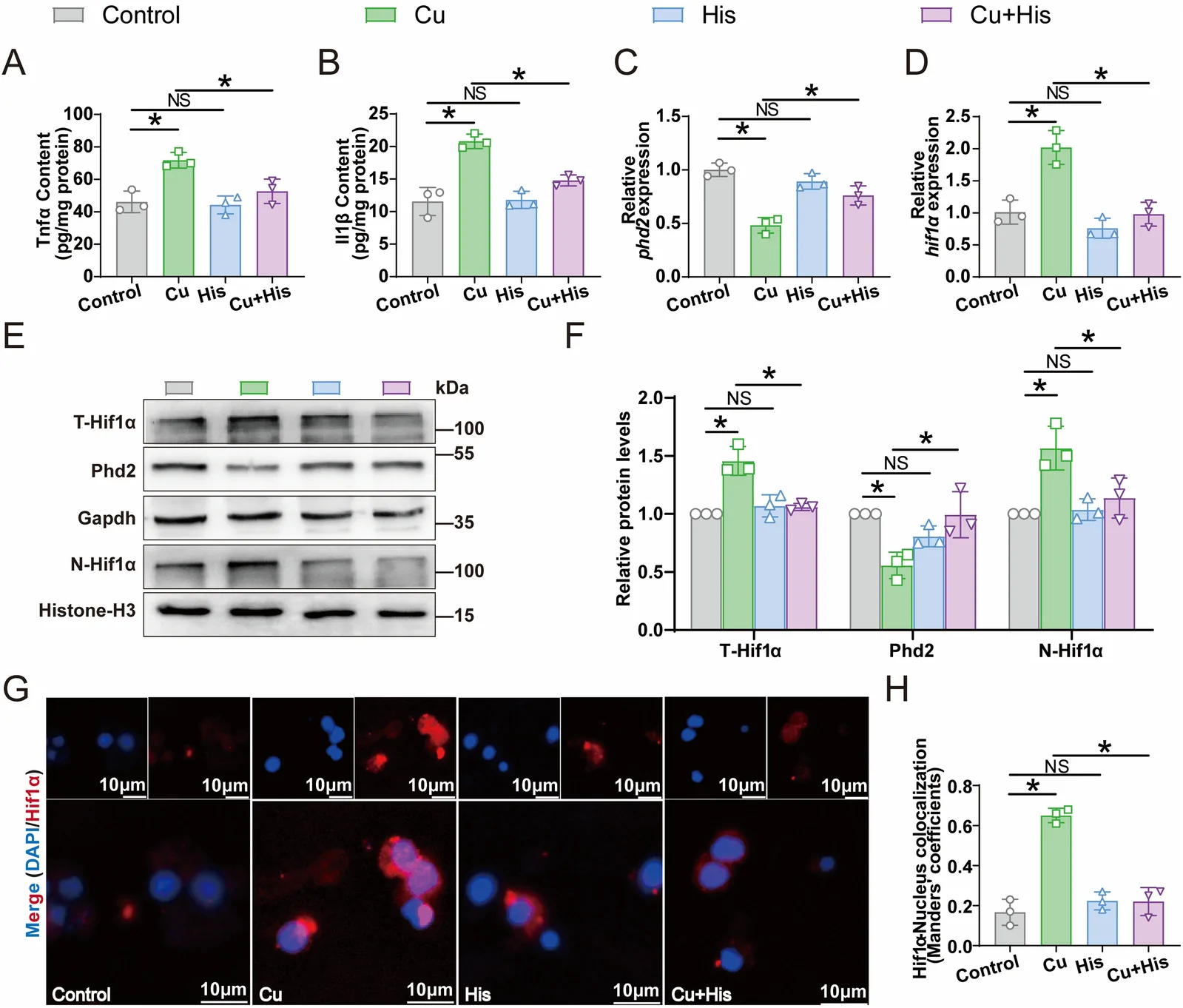
Cu and His regulated Hif1α and inflammation in rainbow trout enterocytes. Isolated rainbow trout enterocytes were subjected to Cu and His for 24 h. **(A)** Tnfα content. **(B)** Il1β content. **(C)** *phd2* mRNA expression. **(D)** *hif1α* mRNA expression. **(E, F)** Western blot analysis of T-Hif1α, Phd2, and N-Hif1α protein expression. **(G)** Immunofluorescence staining showed the typical confocal image and the relative red fluorescence intensity of Hif1α protein. **(H)** Hif1α-to-Nucleus colocalization was quantified as Manders’ coefficients of images obtained in **(G)** Values are means ± S.D. (*n* = 3 independently biological experiments). *p* value was calculated by Student’s t-tests. Asterisk (*) indicate significant difference among two groups (*p < 0.05).

At the gene expression level, Cu significantly downregulated *phd2* and upregulated *hif1α* mRNA versus Control. Cu+His significantly increased *phd2* and decreased *hif1α* mRNA expression compared to Cu (**Figures 6C, D**). Protein analysis confirmed that Cu significantly decreased Phd2 protein while increasing T-Hif1α and N-Hif1α levels relative to Control (**Figures 6E, F**). Cu+His significantly upregulated Phd2 and downregulated T-Hif1α and N-Hif1α protein compared to Cu (**Figures 6E, F**). Immunofluorescence staining further demonstrated that histidine supplementation attenuated the Cu-induced nuclear accumulation of Hif1α (**Figures 6G, H**). Collectively, these *in vitro* results corroborate the *in vivo* findings, demonstrating that histidine supplementation mitigates Cu-induced oxidative stress, mitochondrial dysfunction, inflammation, and nuclear Hif1α accumulation in enterocytes.

#### 3.2.2 Oxidative stress mediates the effects of Cu and histidine on mitochondrial function, Hif1α expression, and inflammation in rainbow trout enterocytes

To investigate whether oxidative stress plays a pivotal role in mediating Cu-induced mitochondrial damage, Phd2-Hif1α pathway dysregulation, and inflammation, primary rainbow trout intestinal cells were pretreated with NAC (5mM) (**Supplementary Figure 2A**). NAC pretreatment significantly attenuated the Cu-induced increases in malondialdehyde (MDA) and reactive oxygen species (ROS) levels (**Figures 7A–C**; **Supplementary Figure 5A**). Concurrently, NAC significantly mitigated the Cu-mediated reductions in the NAD^+^/NADH ratio, ATP content, JC-1 aggregate formation, and mtDNA copy number (**Figures 7D–G**; **Supplementary Figure 5B**). Furthermore, NAC alleviated the Cu-induced downregulation of Phd2 protein expression and the upregulation of both T-Hif1α and N-Hif1α protein levels (**Figures 7H–K**). Finally, NAC pretreatment significantly reduced the Cu-elevated levels of the pro-inflammatory cytokines Tnfα and Il1β (**Figures 7L, M**). These results demonstrate that oxidative stress plays a critical role in mediating Cu-induced mitochondrial dysfunction, Phd2-Hif1α pathway activation, and inflammation. Consequently, the protective effects of histidine supplementation against these pathologies are likely mediated through the modulation of oxidative stress.

**Figure 7:**
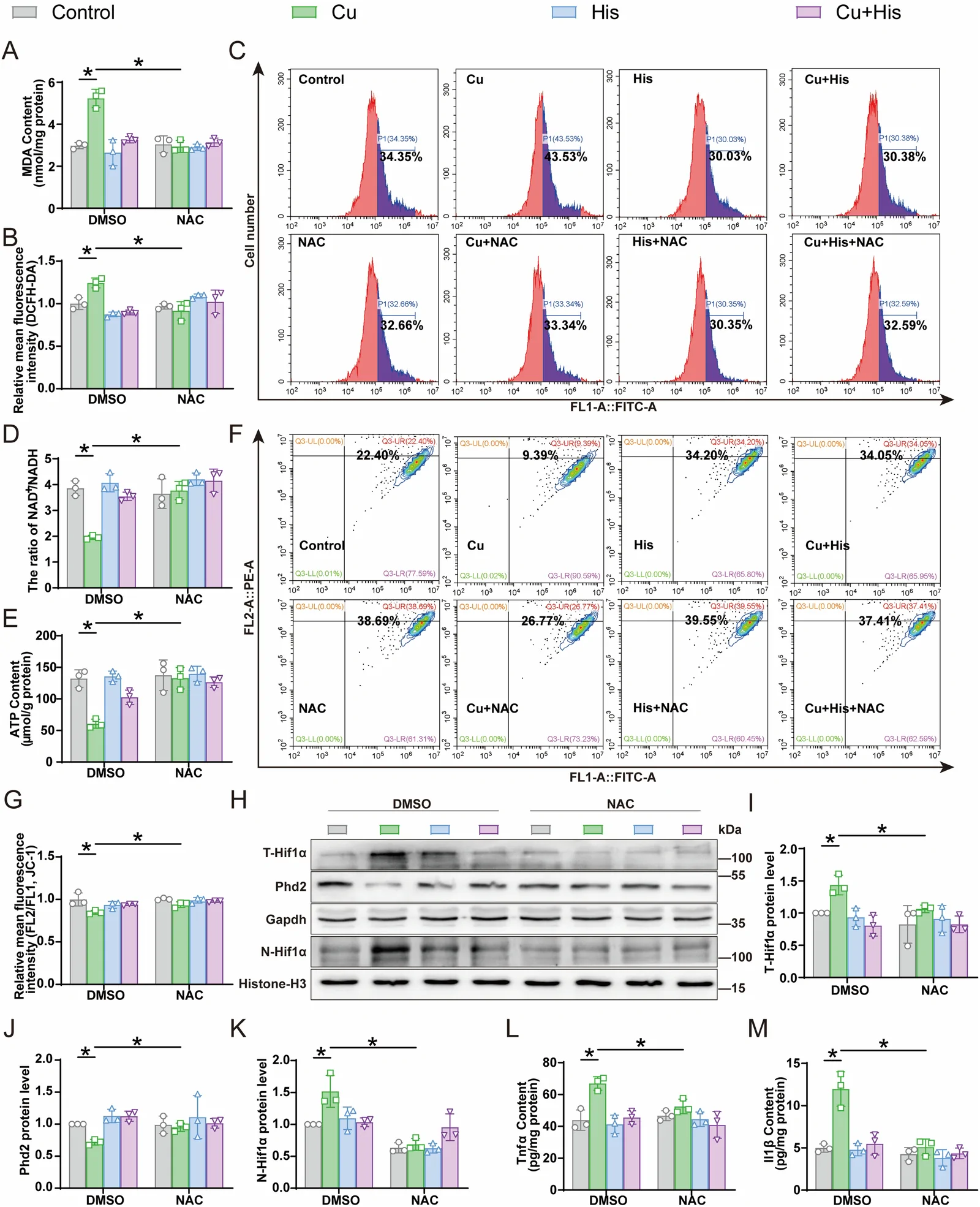
Oxidative stress mediated the changes of Cu and His-induced mitochondrial damage, Hif1α expression, inflammation in rainbow trout enterocytes. Isolated rainbow trout enterocytes were pre-treated with ROS scavenger (5 mM NAC) 2h in advance and then incubated in the control, Cu and His containing medium for 24 h. **(A)** MDA content. **(B)** The intracellular ROS was quantified by calculating FL1 (green) mean fluorescence intensity (DCFH-DA fluorescent staining). **(C)** The fluorescence intensity of ROS was measured by flow cytometry. **(D)** NAD+/NADH ratio. **(E)** ATP content. **(F)** The presence of JC-1 intracellular mitochondrial transmembrane potential (MMP) was determined by flow cytometry analysis of red/green (FL2/FL1) fluorescence ratio. **(G)** The MMP was quantified by flow cytometric analysis of red/green (FL2/FL1) fluorescence ratio (JC-1 fluorescent staining, 5 μM). **(H–K)** Western blot analysis of T-Hif1α, Phd2, and N-Hif1α protein expression. **(L)** Tnfα content. **(M)** Il1β content. Values are means ± S.D. (*n* = 3 independently biological experiments). *p* value was calculated by Student’s t-tests. Asterisk (*) indicate significant difference among two groups (*p < 0.05).

#### 3.2.3 Mitochondrial damage and the Hif1α-Tnfα/Il1β pathway mediate the effects of Cu and histidine on inflammation in rainbow trout enterocytes

To determine if mitochondrial damage mediate the inflammatory effects of Cu and histidine in primary rainbow trout enterocytes, we employed the specific mtROS scavenger (MitoTEMPO, 10 μm) (**Supplementary Figure 3A**). MitoTEMPO incubation significantly attenuated the Cu-induced decreases in the NAD^+^/NADH ratio and ATP content (**Figures 8A, B**). While MitoTEMPO significantly mitigated the Cu-induced elevation of IL1β content, it had no significant effect on Tnfα content (**Figures 8C, D**). These results indicate that mitochondrial damage is involved in the Cu-induced inflammatory response, but cannot fully alleviate this process.

**Figure 8:**
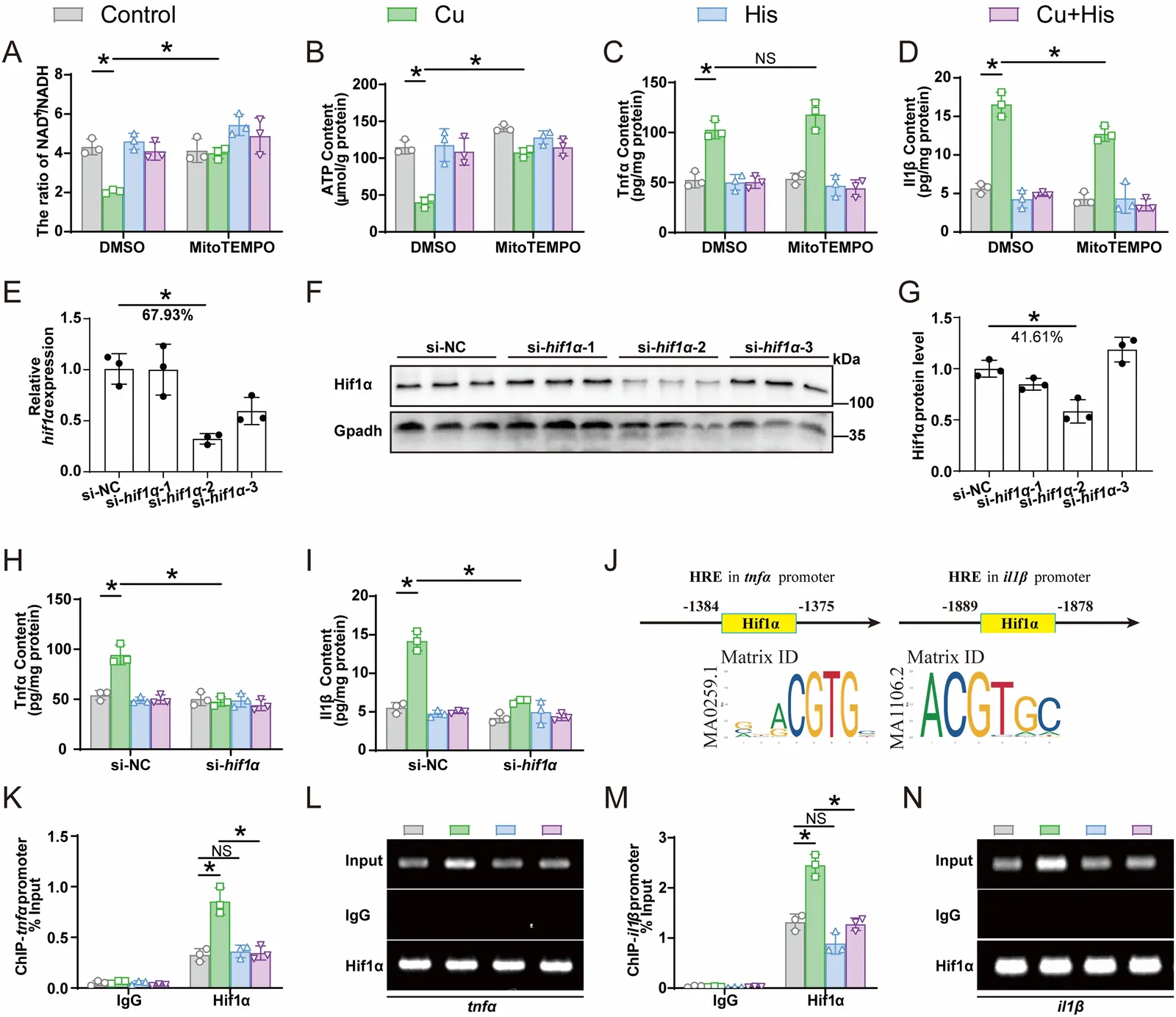
Hif1α-Tnfα/Il1β pathway mediates Cu and His-induced inflammation. Isolated rainbow trout enterocytes were pre-treated with mitochondrial ROS scavenger (10 µM MitoTEMPO) 2h in advance. After transfecting cells with the same amount of negative control siRNA and *hif1α* siRNA, enterocytes were treated with Cu and His for 24 h. **(A)** NAD+/NADH ratio. **(B)** ATP content. **(C)** TNFα content. **(D)** IL1β content. **(E)** mRNA level of *hif1α* gene in Hif1α-knockdown experiment. **(F, G)** Western blot analysis of Hif1α protein level (n = 3). **(H)** TNFα content. **(I)** IL1β content. **(A, J)** The MRE binding sequence *tnfα* (located in -1384 to -1375bp) *and il1β* (located in -1889 to -1878bp) promoter of rainbow trout. Core sequence was predicted from JASPAR. **(K)** ChIP assay for Hif1α binding with *tnfα* promoters by qRT-PCR. **(L)** ChIP assay for Hif1α binding with *tnfα* promoters by RT-PCR. **(M)** ChIP assay for Hif1α binding with *il1β* promoters by qRT-PCR. **(N)** ChIP assay for Hif1α binding with *il1β* promoters by RT-PCR. Values are means ± S.D. (*n* = 3 independently biological experiments). *p* value was calculated by Student’s t-tests. Asterisk (*) indicate significant difference among two groups (*p < 0.05).

Given the established role of HIF1α in regulating oxidative stress and inflammation (42, 43), we investigated its involvement using siRNA-mediated knockdown. Transfection with *sihif1a-2* effectively reduced endogenous *hif1α* mRNA expression (by 67.93%) and protein expression (by 41.61%), establishing it as the optimal construct (**Figures 8E–G**). Knockdown of Hif1α attenuated the Cu-induced increases in Tnfα and Il1β contents (**Figures 8H, I**). As HIF1α regulates gene expression by binding specific promoter sequences (44), we used JASPAR (https://jaspar.genereg.net/) to identify putative Hypoxia-Response Elements (HREs): one within the Tnfα promoter (-1384 to -1375 bp) and one within the Il1β promoter (-1889 to -1878 bp) (**Figure 8J**). ChIP assays confirmed that Cu significantly enhanced Hif1α binding to both the *tnfα* and *il1β* promoters compared to the control. Conversely, Cu+His reduced Hif1α binding to these promoters relative to Cu (**Figures 8K–N**). Collectively, these integrated findings demonstrate that histidine supplementation alleviates Cu-induced activation of the Hif1α-Tnfα/Il1β pathway by reducing the binding affinity of Hif1α within the *tnfα* and *il1β* promoters.

## 4 Discussion

This study reveals that histidine alleviates Cu excess-induced intestinal inflammation through multiple interconnected mechanisms. Key findings demonstrate that histidine attenuates Cu-induced oxidative stress and consequent mitochondrial damage, while simultaneously mitigating the Cu-triggered nuclear accumulation of Hif1α protein. Crucially, histidine reduces inflammation by inhibiting the binding of Hif1α to the promoters of pro-inflammatory genes *tnfα* and *il1β*. Collectively, histidine ameliorates Cu-induced intestinal inflammation by suppressing both oxidative stress-mediated mitochondrial dysfunction and the Hif1α-Tnfα/Il1β signaling pathway. These findings elucidate the intrinsic mechanism by which histidine exerts protective effects against Cu-associated intestine inflammation in rainbow trout. Meanwhile, the rainbow trout model used in this study benefits from its relatively low maintenance cost, rapid life cycle, and the ability to conduct large-scale experiments, providing an excellent model for screening anti-inflammatory substances and evaluating the inflammatory effects of heavy metal toxicity. This research on the interaction between dietary and intestinal immunity has analogical and inspiring value for understanding the possible environmental factors influencing chronic inflammatory diseases in humans.

In this study, dietary Cu excess significantly reduced the FBW, WG, and SGR of rainbow trout. These findings confirm the critical role of Cu in growth performance and demonstrate that elevated Cu concentrations significantly inhibit growth, in agreement with previous findings (2). Concurrently, dietary histidine supplementation effectively mitigated the growth impairment induced by Cu excess. This result indicates that histidine can successfully alleviate the adverse effects of Cu excess in fish, consistent with observations in grass carp (*Ctenopharyngodon idella*) by Jiang et al. (17). This study also confirms that Cu excess causes severe damage to the intestinal villi of rainbow trout. Intestinal villi serve as the core structural units for nutrient absorption, and their morphological integrity constitutes the physiological foundation for normal digestive and absorptive function (45). The disruption of villus structure induced by Cu excess directly indicates impaired intestinal absorption, a phenotype consistent with reports of Cu excess causing intestinal morphological abnormalities in other species (46). Notably, dietary histidine supplementation significantly alleviated the morphological disarray of villi induced by Cu excess, demonstrating that histidine can effectively protect against or repair intestinal structural damage, thereby maintaining absorptive function. Zeng et al. also observed a similar phenomenon (47). Furthermore, Cu excess significantly reduced the number of intestinal goblet cells. The mucus layer secreted by goblet cells is a key physical and chemical barrier protecting the intestine against pathogens, toxins, and mechanical damage (48). A decline in goblet cell numbers severely compromises intestinal barrier function and innate defense capacity, which aligns with existing findings that Cu excess reduces goblet cell abundance (5). Our further experiments confirmed that supplementing histidine to a Cu excess diet reversed the reduction in goblet cells, indicating an antagonistic effect between Cu and histidine in regulating intestinal barrier homeostasis.

Tissue elemental analysis revealed that Cu excess led to a significant increase in intestinal Cu content. Local intestinal Cu homeostasis is a prerequisite for maintaining the activity of Cu-dependent enzymes and cellular functions (41). Dietary Cu excess directly leads to excessive intestinal Cu accumulation and disrupts homeostatic balance, which is a hallmark feature of the Cu overload model. Additionally, we found that Cu excess significantly upregulated the expression of the pro-inflammatory cytokines Tnf-α and Il-1β in the intestine. These cytokines are key mediators of inflammatory cascades and drivers of tissue damage; their elevated expression indicates that Cu excess triggers a robust intestinal inflammatory response (5). Supplementing the Cu excess diet with histidine significantly downregulated the expression of both pro-inflammatory cytokines, confirming that histidine effectively inhibits the activation of inflammation pathways induced by Cu excess. Similarly, Jiang et al. also found that supplementing histidine in feed effectively prevents intestinal damage caused by Cu (17). In summary, Cu excess can induce intestinal inflammation and structural damage by upregulating Tnf-α and Il-1β, whereas supplementation with histidine effectively alleviates this damage process, contributing to a better understanding of the fundamental mechanisms underlying intestinal inflammation.

To investigate how Cu and histidine affect intestinal inflammation, we measured oxidative stress-related indicators. The study revealed that Cu excess significantly increased MDA content and reduced antioxidant enzyme activity, consistent with previous findings on oxidative stress induced by Cu excess (2). Furthermore, supplementation with histidine significantly alleviated these adverse effects by activating the antioxidant defense system to suppress oxidative stress (11). Our results also indicate that under conditions of Cu excess combined with additional histidine, intestinal inflammation is primarily driven by oxidative stress, and pretreatment with NAC effectively mitigates both oxidative stress and intestinal inflammation. These findings support the hypothesis that oxidative stress serves as a key mediator through which histidine alleviates intestinal inflammation caused by Cu.

Mitochondria, as the primary energy-producing centers within cells, play a crucial role in dysfunction during oxidative stress and inflammation (49). Under Cu, mitochondrial swelling, reduced mitochondrial number, significant decreases in NAD^+^/NADH ratio, ATP content, JC-1 fluorescence, and mtDNA copy number indicate severe mitochondrial damage (9). However, histidine supplementation significantly alleviated these impairments, suggesting that histidine can effectively mitigate mitochondrial damage (50). Mitochondrial damage often triggers intestinal inflammation (51). Thus, we further investigated the role of mitochondrial damage in the inflammatory response changes observed in enterocytes under Cu and histidine treatment. Mitochondrial ROS (mtROS) are a central factor driving mitochondrial damage (52); therefore, we treated primary rainbow trout enterocytes with mitochondrial ROS scavenger (MitoTEMPO) to explore the critical role of mitochondrial damage in intestinal inflammation. We found that MitoTEMPO treatment significantly ameliorated the reductions in NAD^+^/NADH ratio and ATP content induced by Cu. This indicates that Cu-induced mitochondrial damage is driven by mtROS, consistent with findings from Zhong et al. (2). Additionally, we observed that MitoTEMPO treatment reduced the increase in Il1β content caused by Cu, although it did not completely eliminate the effects of intestinal inflammation. We believe that this difference is likely due to the inherent and different regulatory mechanisms of these two key pro-inflammatory factors, Tnfα and Il1β. The expression and biological activity of IL1β highly depend on the activation of the NLRP3 inflammasome (53, 54). Meanwhile, mtROS is widely regarded as a potent trigger factor for activating the NLRP3 inflammasome (55). In our study, the clearance of mtROS by MitoTEMPO is likely to specifically interfere with the activation of the NLRP3 inflammasome induced by Cu, effectively blocking the expression of IL1β. In contrast, the transcription and expression of TNFα are mainly strongly regulated by the NF-κB pathway (56). Although ROS can regulate these pathways, Cu is likely to strongly and dominantly activate these pathways through mtROS-independent mechanisms, resulting in the production of Tnfα. Therefore, inhibiting mtROS is not sufficient to significantly affect this dominant Tnfα induction pathway. Additionally, the transcription factor binding regions of the promoters of TNFα and IL1β genes, the sensitivity thresholds to oxidative stress, and the complex regulatory networks between them also differ (57).

Hif1α, a heterodimeric helix-loop-helix transcription factor, is a crucial mediator in the cellular response to oxidative stress (58). We observed that Cu significantly elevated the protein levels of both T-Hif1α and N-Hif1α. Previous study has also found that Cu can upregulate HIF1 expression (59). Our study reveals that, mechanistically, Cu primarily suppresses Phd2 protein expression through oxidative stress, thereby promoting the nuclear translocation of Hif1α. This mechanism is supported by the effects of the ROS scavenger NAC. Concurrently, histidine supplementation significantly inhibited Hif1α expression, indicating an antagonistic effect between histidine and Cu. Supporting evidence shows that histidine can directly act as a ROS scavenger, protecting cells from oxidative damage (60). Furthermore, Aschner et al. have emphasized that HIF1α activation is inextricably linked to ROS levels and PHD2 expression (61). Existing evidence confirms that oxidative stress inhibits Phd2 activity, leading to Hif1α activation (19). However, although our study implicates oxidative stress as the key mediator through which Cu and histidine influence Hif1α, the specific mechanisms underlying its role in intestinal inflammation remain incompletely characterized.

In order to gain deeper insight into the role of Hif1α in inflammatory responses, we analyzed its interaction with Tnfα and Il1β. We first observed that Hif1α knockdown significantly alleviated the increase in Tnfα and Il1β contents induced by Cu, indicating an inseparable relationship between Hif1α and Tnfα/Il1β. Numerous studies have also shown that Hif1α and Tnfα/Il1β form a tightly coupled signaling network, often coexisting and mutually reinforcing each other (62, 63). Subsequently, immunofluorescence and ChIP assays confirmed that Hif1α binds to the promoter regions of both the *tnfα* and *il1β* genes. Notably, ChIP results demonstrated that Cu enhances the binding activity of Hif1α to the *tnfα* and *il1β* promoters, while histidine supplementation alleviated this effect. Wu et al. also found that Cu regulates the transcriptional activity of HIF1α by influencing its selective binding to the promoters of target genes (64). However, the existing evidence suggests that Hif1α participates in the regulatory process by binding to these regions, but the specific function of the HRE site still needs to be further verified. These findings establish a direct transcriptional regulatory relationship between Hif1α and *tnfα*/*il1β*: Cu promotes the nuclear translocation of Hif1α, which subsequently activates the transcription of *tnfα* and *il1β*, ultimately leading to intestinal inflammatory injury. Conversely, histidine supplementation effectively counteracts this pathological process.

## 5 Conclusion

In summary, histidine can reverse Cu-induced mitochondrial damage and also affects the binding of Hif1α to the promoters of the *tnfα* and *il1β* genes, thereby alleviating intestinal inflammation. The underlying regulatory mechanism is as follows: Cu induces excessive activation of intestinal oxidative stress, which on one hand causes damage to mitochondrial structure and function, and on the other hand mediates sustained activation of the Hif1α-Tnfα/Il1β signaling pathway; supplementation with histidine effectively blocks this pathological process (**Figure 9**). However, this study still has certain limitations. The research was conducted only on rainbow trout through *in vivo* and *in vitro* validations, and the related molecular regulatory mechanisms were specific to the fish species. They do not yet have the conditions to be directly extrapolated to other aquatic organisms or even terrestrial animals for clinical treatment of intestinal inflammation. In conclusion, This study systematically delineates the molecular regulatory network through which Cu induces intestinal inflammation, confirms mitochondrial damage and Hif1α as the core regulatory nodes mediating this pathology, and provides a theoretical basis for Hif1α as a potential therapeutic target for intestinal inflammation intervention.

**Figure 9:**
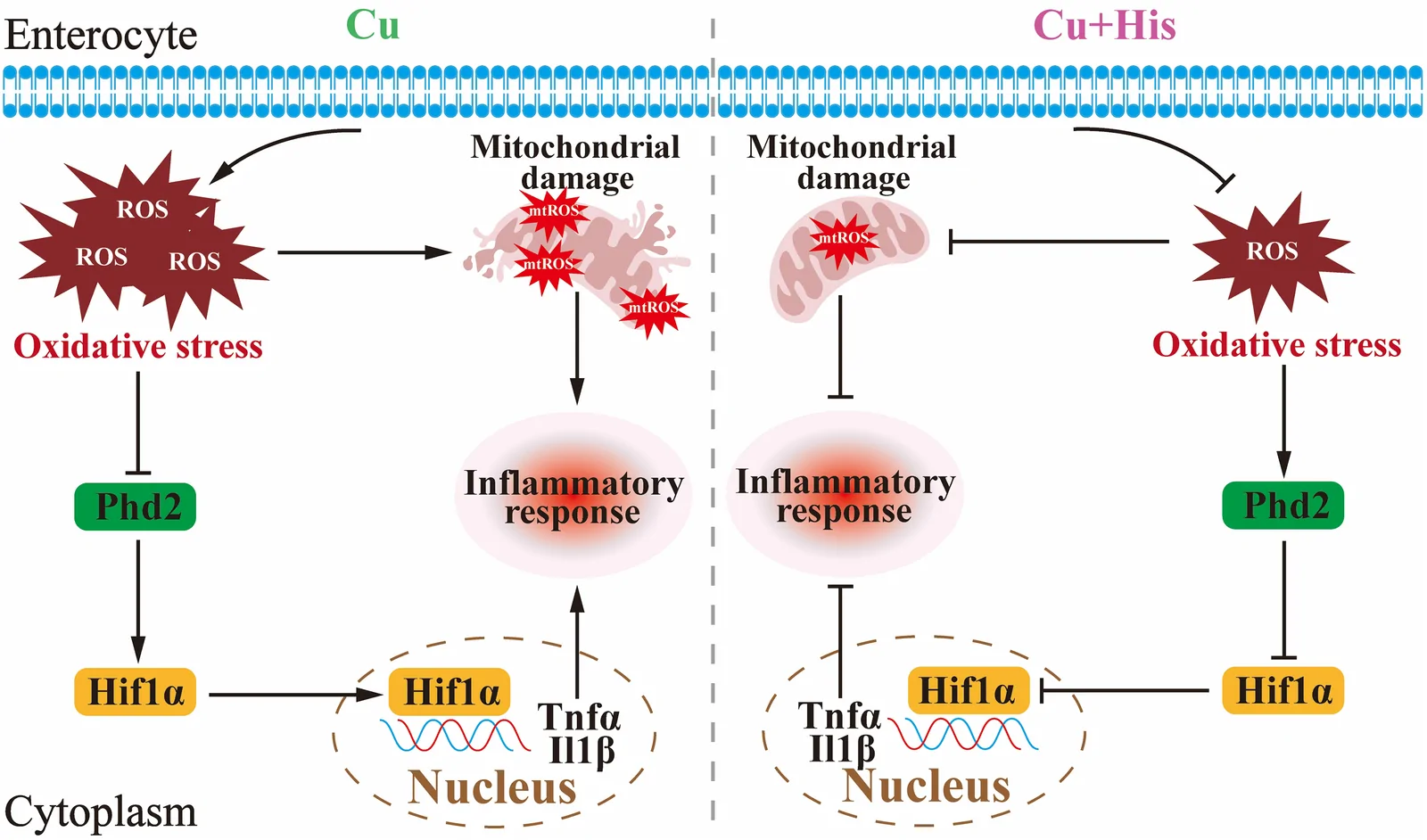
The mechanistic model of histidine ameliorating intestinal inflammatory responses induced by Cu.

## Data Availability

The original contributions presented in the study are included in the article/**Supplementary Material**. Further inquiries can be directed to the corresponding author/s.
