# Supplementary material for: Histidine alleviates copper-induced intestinal inflammation through oxidative stress-mediated mitochondrial damage and the Hif1α-Tnfα/Il1β pathway
Source: Front Immunol. 2026 Oct 5;17:1960076. doi: 10.3389/fimmu.2026.1960076 (PMC13677363; doi:10.3389/fimmu.2026.1960076)
Supplement: Supplementary file 1 [file DataSheet2.pdf]

frontiers

The International Society for the Advancement of Cytometry (ISAC) has highlighted the importance of including comprehensive methodological information to ensure data reproducibility and reliability. In line with this, Frontiers in Immunology now requires authors to submit a checklist for manuscripts that involve flow or mass cytometry. This checklist helps standardize the reporting process, improving the quality and transparency of published data. By doing so, we support scientific progress, making it easier for other researchers to replicate and validate experiments.

This form should be submitted with any manuscripts using flow or mass cytometry.

Sample/specimen/material description

☐ Total blood

☐ PBMCs

☐ Organ digests

Other Primary intestinal epithelial cells (IECs) isolated from rainbow trout

Did the samples suffer any treatment before or after incubation with the antibodies?

☐ Drug ________________________________

☐ Cell permeabilization ____________________

☐ Dye __________________________________

☐ Propidium iodine

☑ Not applicable

Other ___________________________________

Instrument and antibodies

Name of the Cytometer CytoFlex flow cytometer (Beckman Coulter)

Antibodies and targets	Fluorochrome/ Metal	Catalog number/Company
e.g. anti-CD4	FITC	Cat. XXX/ XXX ltd.
JC-1 fluorescent probe	FITC and PE	Beyotime Biotech, C2006

DCFH-DA fluorescent probe	FITC	Beyotime Biotech, S0033M

Data analyses

1. Name of the software FlowJo v.10
2. Reference gating strategy in the manuscript or supplementary material

Gating strategy in (eg Figure X) Supplementary Figure 4 and 5
