# Supplementary material for: Histidine alleviates copper-induced intestinal inflammation through oxidative stress-mediated mitochondrial damage and the Hif1α-Tnfα/Il1β pathway
Source: Front Immunol. 2026 Oct 5;17:1960076. doi: 10.3389/fimmu.2026.1960076 (PMC13677363; doi:10.3389/fimmu.2026.1960076)
Supplement: Supplementary file 2 [file DataSheet1.doc]

Histidine alleviates copper-induced intestinal inflammation through oxidative stress-mediated mitochondrial damage and the Hif1α-Tnfα/Il1β pathway

## Chongchao Zhong^a^, Fang Chen^a^, Shaoxia Lu^a^, Shicheng Han^a^, Hongbai Liu^a,*^, Changan Wang^a,*^

^a^Key Laboratory of Aquatic Animal Diseases and Immune Technology of Heilongjiang Province, Heilongjiang River Fisheries Research Institute, Chinese Academy of Fishery Sciences, Harbin 150000, Heilongjiang Province, China

***** Authors to whom correspondence should be addressed.

**Email address:** liuhongbai@hrfri.ac.cn (H. Liu). wangchangan@hrfri.ac.cn (C. Wang),

**Supplementary Figure 1. Concentration screening of Cu and Histidine.** The rainbow trout enterocytes were incubated in the control, Cu, and His containing the medium for 24h. (A) Cell viability after Cu incubation. (B) Cell viability after His incubation. (C) Cell viability after Cu+His co-incubation. All data were expressed as mean ± S.D. (n=3 independently biological experiments). Different letters (a-d) indicate significant difference among three treatments (one-factor ANOVA, and further post hoc Duncan’s multiple range test. *P* ＜ 0.05). *P* value was calculated by Student’s t tests. Asterisk (*) indicated significant difference between two groups (P< 0.05).

**Supplementary Figure 2. Concentration screening of ROS scavenger.** The rainbow trout enterocytes were incubated in the control and ROS scavenger (NAC) containing the medium for 24h. (A) Cell viability after NAC incubation. All data were expressed as mean ± S.D. (n=3 independently biological experiments). P value was calculated by Student’s t tests. Asterisk (*) indicated significant difference between two groups (P< 0.05).

**Supplementary Figure 3. Concentration screening of mitochondrial ROS scavenger.** The rainbow trout enterocytes were incubated in the control and mitochondrial ROS scavenger (mitoTEMPO) containing the medium for 24h. (A) Cell viability after incubation. All data were expressed as mean ± S.D. (n=3 independently biological experiments). Different letters (a-c) indicate significant difference among three treatments (one-factor ANOVA, and further post hoc Duncan’s multiple range test. *P* ＜ 0.05).

**Supplementary Figure 4. Gating strategy.** Isolated rainbow trout enterocytes were subjected to Cu and His for 24 h. (A-B) Gating strategy.

**Supplementary Figure 5. Gating strategy.** Isolated rainbow trout enterocytes were pre-treated with ROS scavenger (5 mM NAC) 2h in advance and then incubated in the control, Cu and His containing medium for 24 h. (A-B) Gating strategy.

**Supplementary Table 1. Formulation and proximate composition analysis (% of dry weight basis) of the basal diet.**

| Ingredients (g kg^-1^) | Control | Cu | His | Cu+His |
| --- | --- | --- | --- | --- |
| Fish meal | 100 | 100 | 100 | 100 |
| Cottonseed protein concentrate | 350 | 350 | 350 | 350 |
| ^1^Compound amino acids (His-Free) | 130 | 130 | 130 | 130 |
| Vital wheat gluten | 100 | 100 | 100 | 100 |
| Wheat flour | 140 | 140 | 140 | 140 |
| Ca（H_2_PO_4_）_2_·H_2_O | 10 | 10 | 10 | 10 |
| Choline chloride | 1 | 1 | 1 | 1 |
| Ascorbyl-2-polyphosphate | 5 | 5 | 5 | 5 |
| ^2^Mineral premix (Cu-Free) | 5 | 5 | 5 | 5 |
| ^3^Vitamin premix | 5 | 5 | 5 | 5 |
| Fish oil | 70 | 70 | 70 | 70 |
| Soybean oil | 70 | 70 | 70 | 70 |
| Cellulose | 5.992 | 5.96 | 5.992 | 5.96 |
| CuSO_4_·5H_2_0 (g) | 0.008 | 0.04 | 0.008 | 0.04 |
| L-Histidine | 4 | 4 | 8 | 8 |
| Glycine | 4 | 4 | 0 | 0 |
| Proximate analysis | | | | |
| Moisture (%) | 6.59 | 6.21 | 6.27 | 6.47 |
| Crude protein (%) | 38.05 | 37.99 | 38.14 | 38.03 |
| Lipid (%) | 17.17 | 17.04 | 17.14 | 17.05 |
| Ash (%) | 3.14 | 3.31 | 3.12 | 3.27 |
| Histidine content (g kg^-1^) | 6.89 | 6.71 | 10.94 | 10.87 |
| Cu content (mg kg^-1^) | 3.11 | 12.69 | 3.24 | 12.37 |

^1^Compound amino acids (g mixture): Lysine, 100.0 mg; Arginine, 115 mg; Valine, 100 mg; Isoleucine, 62 mg; Proline, 66 mg; Phenylalanine, 68 mg; Aspartic acid, 60 mg; Tyrosine, 62 mg; Alanine, 62 mg; Cysteine, 12 mg; Glutamic acid, 76 mg; Leucine, 62 mg; Glycine, 55 mg; Methionine, 50 mg; add with cellulose to a total of 1g.

^2^Minerals premix (g mixture): MgSO4·7H2O, 400 mg; KCl, 300 mg; FeSO4·7H2O, 100 mg; MnSO4·4H2O, 20 mg; ZnSO4·7H2O, 30 mg; KI, 0.6 mg; NaCl, 100 mg; CoCl2, 1 mg; Na2SeO3, 0.6 mg; add with cellulose to a total of 1g.

^3^Vitamin premix (g mixture): vitamin C, 20 mg; vitamin E, 12 mg; vitamin K3, 1 mg; vitamin A, 3000 IU; vitamin D3, 600 IU; vitamin B1, 3 mg; vitamin B2, 6 mg; vitamin B6, 3 mg; vitamin B12, 0.1 mg; nicotinic acid, 35 mg; folic acid, 1 mg; inositol, 200 mg; biotin, 0.5 mg; calcium pantothenate, 10 mg; add with cellulose to a total of 1g.

**Supplementary Table 2.** Primers used for quantitative real-time PCR analysis, siRNA and ChIP analysis.

| **GENES（Rainbow trout）** | **FORWARD PRIMER (5’-3’)** | **REVERSE PRIMER (5’-3’)** | **ACCESSION NO.** |
| --- | --- | --- | --- |
| ***β-actin*** | AGGGAGTGATGGTTGGGATG | CTGTTGGCTTTGGGGTTGAG | NM_001124235.1 |
| ***rpl7*** | ACTTCAAGCCTGCCAACAAC | CATCCTCCTGACGAGTCTGTT | NM_001165156.1 |
| ***hprt*** | CGATAAGTCTGTCCCGTTGA | CATAGTCCTCCCAGTCTCCAC | XM_036976657.1 |
| ***tuba*** | GGCAACATCAAGACAAAGCG | TTGGCATACATCAGGTCAAACT | XM_036951347.1 |
| ***ubce*** | ACACTCAGGAGATCAGACGCTG | GTTTTCGGAAGGGGCACCAC | XM_021579932.2 |
| ***tbp*** | ACTTGGGCTGTAAACTTGACTT | GGGACTGTTCTTCACTTTTGG | XM_021581318.2 |
| ***gapdh*** | GACACCCACTCCTCCATCTT | GGTTGCTGTAGCCAAACTCA | NM_001124209.1 |
| ***18s rrna*** | AACGGCTACCACATCCAAGG | CCCGAGATCCAACTACGAGC | XR_005053028.1 |
| ***tnfα*** | CTGAAGACATGGAGAGGGGC | ACGGTGTCAGCGGAAAGATT | NM_001124357.1 |
| ***il1β*** | GAAACCATGGTGCTACGTGC | TCGAAGGTGGTCAGGGAGAT | XM_021601282.2 |
| ***il6*** | GTCTGCAGGAGATCAGTCGG | GTCTTTGCCCCTCTTTCCCA | DQ866150.1 |
| ***il8*** | AGAATGTCAGCCAGCCTTGT | TGTTGGCCAGCATCTTCTCA | NM_001124362.1 |
| ***il10*** | ATGTCTCCCTGCTCTCTCCT | GCTCGTGGAAGATGTTTCCG | NM_001245099.1 |
| ***tgfβ*** | GTGACACCATGTCCACCTGT | GCAGTCGATCAGTCCCCAAT | XM_021591332.2 |
| ***sod1*** | TGGCAATGAAGGCTGTTTGC | ACCCGAGATCTCCCCAATCA | NM_001124329.1 |
| ***sod2*** | CTCCCTGACCTGACCTACGA | GCTGCATGATCTCTGCGTTG | XM_021612540.2 |
| ***cat*** | TGTCACACTTCGACCGTGAG | CTCAAACACCTTGGCCTTGC | XM_021564294.2 |
| ***gpx1*** | GACCTGACAGCAAAGCTCCT | GGTGTAATCCCTGGTGGTCG | NM_001124525.1 |
| ***gr*** | TGGCGGACATGTTCACTTCA | CTATGACGCTGCGCTTAGGA | XM_021561547.2 |
| ***mtf-1*** | TCAAGACAAGCCCCAGACAC | TGGTAATAGACGGATGCGGC | XM_021559481.2 |
| ***nrf2*** | AGGAGGAGGAGAAGAGGCAG | GCCAGCAGCTGCATACATTC | XM_021597223.2 |
| ***hif1α*** | CCTCCATCATGAGACTGGCC | CATCCGCAAGAAGAAGCTGC | AF304864.1 |
| ***hsf1*** | CCCAAGTTCAGCAGGCAGTA | CAGGGGAGTGTCTACCTGGA | XM_021559489.2 |
| ***foxo1*** | CAACAACAGCAAGTTCGCCA | GGCCTAGAGAACTCGCCATC | XM_036965061.1 |
| ***foxo3*** | CCCAACCACCTCTCCCTCTA | CGTGGTCCCAAAGGTCATCA | XM_021600701.2 |
| ***phd2*** | CCCGCAAAAACTGGCTATGG | CGCCGTTAGGGTTATCCACA | XM_021557379.2 |
| ***atp6*** | CCCACTTATCGCCGTAGCAT | AGGTTGGTTTCGTATGCCGA | L29771.1 |
| ***eef1a1*** | GTGACAACGTGGGCTTCAAC | CACTGCAAAACGACCCAGTG | XM_021610662.2 |
| **ChIP-**  **qPCR** | **Forward primer (5’-3’)** | **Reverse primer (5’-3’)** |  |
| ***tnfα*** | TGATGAAACCAAGATGGAACTC | ATGCTTCACCGTAGGGATGG |  |
| ***il1β*** | CGTCTCGTCGTTGTTGGTAA | TGTGGAAGAACTTGACTGGACT |  |
| **siRNA** | **Sense(5'-3')** | **Antisense(5'-3')** |  |
| **si*hif1α*-1** | GGGUUAUGAUCCAGAGGACTT | GUCCUCUGGAUCAUAACCCTT |  |
| **si*hif1α*-2** | GAGGAUAUGAGGCCUGUGATT | UCACAGGCCUCAUAUCCUCTT |  |
| **si*hif1α*-3** | GACAUCAAUGCUGAGUUUATT | UAAACUCAGCAUUGAUGUCTT |  |

**Abbreviations**: *18s rrna, 18S ribosomal RNA; atp6, ATP synthase F0 subunit 6; β-actin, beta-actin; cat, catalase; eef1a1, eukaryotic translation elongation factor 1 alpha 1; foxo1, forkhead box O1; foxo3, forkhead box O3; gapdh, glyceraldehyde-3-phosphate dehydrogenase; gpx1, glutathione peroxidase 1; gr, glutathione reductase; hif1α, hypoxia-inducible factor 1 alpha; hprt, hypoxanthine phosphoribosyltransferase; hsf1, heat shock factor 1; il1β, interleukin 1 beta; il6, interleukin 6; il8, interleukin 8; il10, interleukin 10; mtf-1, metal regulatory transcription factor 1; nrf2, nuclear factor erythroid 2-related factor 2; phd2, prolyl hydroxylase domain protein 2; rpl7, ribosomal protein L7; sod1, superoxide dismutase 1 (Cu/Zn-SOD); sod2, superoxide dismutase 2 (Mn-SOD); tbp, TATA-box binding protein; tgfβ, transforming growth factor beta; tnfα, tumor necrosis factor alpha; tuba, tubulin alpha chain; ubce, ubiquitin-conjugating enzyme.*
